# Contextual interference in children with brain lesions: a pilot study investigating blocked vs. random practice order of an upper limb robotic exergame

**DOI:** 10.1186/s40814-021-00866-4

**Published:** 2021-06-25

**Authors:** Judith V. Graser, Caroline H. G. Bastiaenen, Anja Gut, Urs Keller, Hubertus J. A. van Hedel

**Affiliations:** 1 Research Department, Swiss Children’s Rehab, University Children’s Hospital Zurich, Mühlebergstrasse 104, 8910 Affoltern am Albis, Switzerland; 2 Children’s Research Centre CRC, University Children’s Hospital Zurich, University of Zurich, Zurich, Switzerland; 3 Research Group Function, Participation and Rehabilitation CAPHRI, Department of Epidemiology, Maastricht University, Maastricht, the Netherlands

**Keywords:** Feasibility, Vanguard trial, Paediatric rehabilitation, Robotic exergames, Variable practice, Blocked versus random order

## Abstract

**Introduction:**

Evidence about contextual interference in children with brain lesions when practising motor tasks is lacking. Our main objective was to evaluate the feasibility of a randomised controlled trial (RCT) comparing blocked with random practice order of an upper limb robotic exergame to improve reaching in children with neuromotor disorders with a pilot trial.

**Methods:**

We recruited children with brain lesions and impaired upper limb functions who underwent a 3-week schedule that consisted of baseline assessments, intervention period (participants were randomised to a blocked or random order group), and follow-up assessment. We evaluated ten feasibility criteria, including the practicability of the inclusion/exclusion criteria, recruitment rate, feasibility of randomisation, scheduling procedure, and the participants’ programme adherence.

**Results:**

The inclusion/exclusion criteria were not completely feasible as patients who were not able to perform the exergames were included. Twelve participants were recruited, and six datasets were used for analysis. The scheduling and randomisation procedures were generally feasible, but the procedure was only partially feasible for the participants, as some sessions were aborted due to lack of motivation and fatigue.

**Conclusion:**

An RCT following this study protocol is not feasible. We formulated suggestions for future studies that aim to investigate contextual interference as in this pilot study.

**Trial registration:**

ClinicalTrials.gov Identifier: NCT02443857, registered on May 14, 2015

**Supplementary Information:**

The online version contains supplementary material available at 10.1186/s40814-021-00866-4.

## Introduction

Motor learning forms the basis of neurorehabilitation [1]. The initial phase during which motor skills are gained is referred to as acquisition. Nevertheless, it is important for successful neurorehabilitation that a practised motor skill can be maintained over time and generalised to another setting or situation. Hence, the aspects of motor learning that are of particular interest for neurorehabilitation are retention and transfer. We need a better understanding of motor learning to integrate underlying principles such as different types of practice or feedback successfully in, for example, hand rehabilitation [2].

Motor learning requires practice, consisting of a high number of repetitions but not of the exact same movement [3], as practice should include some variability (‘repetition without repetition’ [4]). It has been proposed that motor learning is generated when motor problems need to be solved [2]. Considering this, ‘repetition without repetition’ is not repeating the solution for a specific motor problem but repeating the process of solving the problem by improving it with every trial [4]. Performing a large number of repetitions can be tedious, especially for children. Hence, another reason for implementing variable practice is to influence the motivation needed to perform so many repetitions constantly in a positive way.

Variable practice is understood as practising different variations of the task. An effect occurring when practising different tasks within one therapeutic session is contextual interference [5]. The contextual interference effect in motor learning refers to the interference that results from practising a task within the context of other tasks in a practice session. A continuum of increasing variability leads to different levels of contextual interference. When variability is low, for example, when tasks are practised in blocked order, i.e. several repetitions of one task before switching to the next task (e.g. AAA…, BBB…, CCC…), the level of contextual interference is low [6]. When variability is high, for example, if tasks are performed in random order (e.g. ABC…, CCA…, BAB…), participants practise under conditions of high contextual interference [6]. In healthy adults, practising with low contextual interference leads to improved acquisition but reduced transfer and retention compared to practising with high contextual interference [6].

Three different hypotheses underlying this effect have been discussed: the elaboration hypothesis, the forgetting-reconstruction hypothesis, and the retroactive inhibition hypothesis. The elaboration hypothesis suggests that practising skills in random order results in more distinctive processing and is beneficial for transfer and retention [7, 8]. The forgetting-reconstruction hypothesis is based on the idea that practising in random order requires the learner to forget after each task what he or she learned in order to focus on the next task [8]. This might disturb performance during acquisition but is beneficial for retention [8]. The retroactive inhibition hypothesis [9–11] suggests that the contextual interference effect occurs due to a disadvantage of blocked practice and not due to an advantage of random practice. Blocked practice order might inhibit the previous memory leading to differences in performance between the random and blocked practice [10].

In children and youths practising typical sports skills in a field setting under high contextual interference (random) or mixed (random and blocked) conditions, a systematic review and meta-analysis reported effect sizes between −0.8 and 0.3 for transfer or retention measures [12]. However, the difference between blocked and random practice order was not evaluated. Another systematic review included 25 papers investigating contextual interference in typically developing children and children with brain lesions [13]. No conclusion about the contextual interference effect could be made as results were diverging, the methodological quality of these studies was low, and the risk of bias high [13].

In recent years, rehabilitation technologies, such as robot-assisted training devices, have been applied increasingly, particularly in paediatric neurorehabilitation [14, 15]. Robot-assisted devices in combination with exergames provide playful exercise, which increases engagement and motivation to achieve larger numbers of movement repetitions [16]. Most rehabilitation technologies contain sensors that can be used to estimate the status and functional improvement of patients objectively [17]. Applying such technologies to investigate contextual interference in children with brain lesions seemed therefore a promising option. One recent study applied a computer maze game and compared constant practice order (total of 30 trials of one maze) with random practice order (total of 30 trials of five different mazes) in children with cerebral palsy (CP) and typically developing (TD) children [18]. The random order group performed the maze games faster at retention (TD and CP combined) and transfer (CP and TD combined but also the single groups) [18]. Usually, contextual interference studies include blocked and random order groups that practice the same number of repetitions of each task. This study used constant and random practice order, and group differences might be related to the number of different mazes practised in the two groups (one vs. five) and not the practice order per se. Hence, scientific experience in this specific field remains scarce.

It might be difficult to conduct such a study within a clinical setting for various reasons, including restricted numbers of eligible participants. To avoid unnecessary waste of resources, it is recommended to perform a pilot study preceding the final randomised clinical trial (RCT) [19]. Therefore, our main objective was to evaluate the feasibility of a randomised controlled intervention trial assessing the contextual interference effect by comparing blocked with random order practice of an exergame played with a robotic upper-limb exoskeleton device to improve reaching movements in children with neuromotor disorders.

## Methods

We designed this randomised controlled pilot study with two intervention arms in line with the Consolidated Standards of Reporting Trials (CONSORT) statement extension for randomised pilot and feasibility trials [20], the recommendations about conducting a pilot study provided by Thabane et al. 2010, and the Standard Protocol Items: Recommendations for Interventional Trials (SPIRIT) [21]. Methodological details were described in the study protocol [22].

### Participants

We included children meeting the following inclusion criteria:

- Age between five and 18 years
- Congenital or acquired (subacute or chronic state, i.e. at least 3 months after the incident) brain lesion
- Uni- or bilaterally impaired upper limb function (spasticity, dyskinesia, or mixed conditions)
- Undergoing inpatient neurorehabilitation
- Manual Ability Classification System (MACS) level I (objects are handled easily and successfully) to IV (a limited selection of easily managed objects are handled in adapted situations) [23]
- Ability to sit for approximately 60 min without lateral trunk support
- Ability to understand and follow test instructions
- Ability to visually follow events on a screen in approximately 1 m distance
- Ability to communicate discomfort or pain

Children were excluded if meeting the following exclusion criteria:

- Surgery or upper limb Botox injections during the past  six months
- Upper limb skin lesions
- Severe obesity
- Severe spasticity (Modified Ashworth Scale >3, i.e. considerable increase in muscle tone, passive movement difficulty, rigidity [24])
- Fixed contractures or deformities of the upper limbs.

### Recruitment

Each week, a therapist screened the patients entering the Swiss Children’s Rehab of the University Children’s Hospital Zurich in Affoltern am Albis, Switzerland. Potential participants were informed about the study. Written informed consent was obtained from all the participants’ legal representatives and participants aged 15 years and older, who agreed to participate. All participants provided verbal consent. This study was approved by the Ethics Committee of the Canton of Zurich (BASEC-Nr. PB_2016-02450, subproject 5 motor learning) and the Swiss Agency for Therapeutic Products (Swissmedic reference number: 2015-MD-0009). We enrolled participants for approximately 1 year, between the end of June 2018 and June 2019.

### Group allocation

Participants were allocated to one of the two intervention groups (blocked or random practice order) by randomisation through minimisation, which is a method ensuring the balance of several prognostic factors between study groups in small samples [25]. Minimisation parameters were the following descriptors: age (preschool age: 5–6 years, primary school age: 7–12 years, and secondary school age and older: 13–18 years); gender (female, male); diagnosis (congenital, acquired), manual ability (MACS levels I, II, III, or IV), and cognitive ability according to the Test of Nonverbal Intelligence – Fourth edition (TONI-4), which was applied by a trained neuropsychologist and evaluates abstract reasoning and problem-solving (Index Score <70: very poor, 70–79: poor, 80–89: below average, 90–110: average, 111–120: above average, 121–130: superior, and >130: very superior) [26].

When the principal investigator obtained these data, she informed the study nurse that the data were ready for the minimisation process. Minimisation is a secure method when conducted by an independent person [25]. Therefore, the study nurse person who performed the randomisation and allocation did not participate in any other study procedure and was unaware of the aim of the study. She added the information in the custom-made MATLAB script. In case of absence, a backup person was assigned, who was not involved in other study operations. Participants could not influence the choice in which group they would practice.

### Robot

**Fig. 1:**
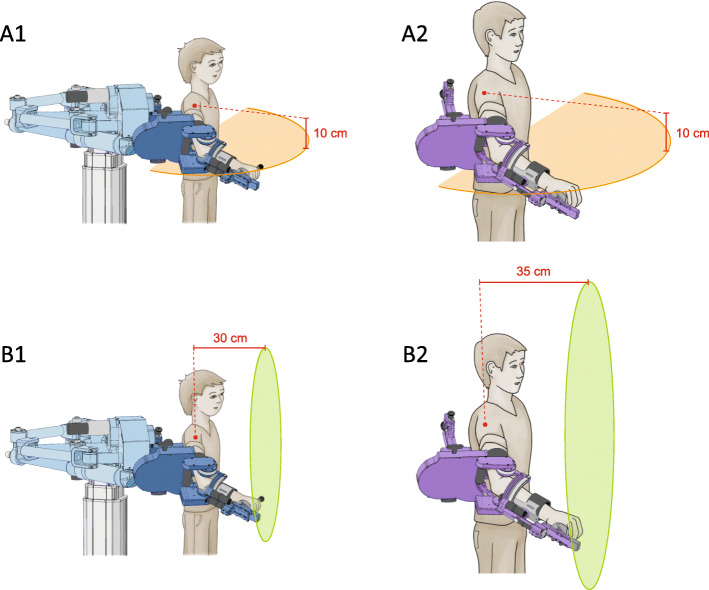
ChARMin with two distal modules and different movement planes. A1 Small distal module (dark blue), movements restricted to the transversal plane. A2 Large distal module (purple), movement direction restricted to the transversal plane. B1 Small distal module (dark blue), movements restricted to the frontal plane. B2 Large distal module (purple), movements restricted to the frontal plane

For this pilot study, we chose the ChARMin robot [27]. In short, ChARMin is an exoskeleton robot for the rehabilitation of upper limb function in children from 5 to 18 years. It can be adjusted to the anthropometric sizes of younger and older children as two different distal modules were developed (Fig. 1). ChARMin can actively support arm movements while playing various audio-visual exergames shown on the screen in front of the participant.

### Exergames

Contextual interference is assumed to occur when different motor programmes are involved in different motor tasks [5]. It has been proposed that spatial and topographical characteristics are invariant within the same motor programmes [4]. Thus, choosing different spatial characteristics (i.e. moving in different planes) ensures the involvement of different motor programmes. Therefore, we designed a specific ChARMin exergame, which can be played in the frontal or the transversal plane (Fig. 1).

**Fig. 2:**
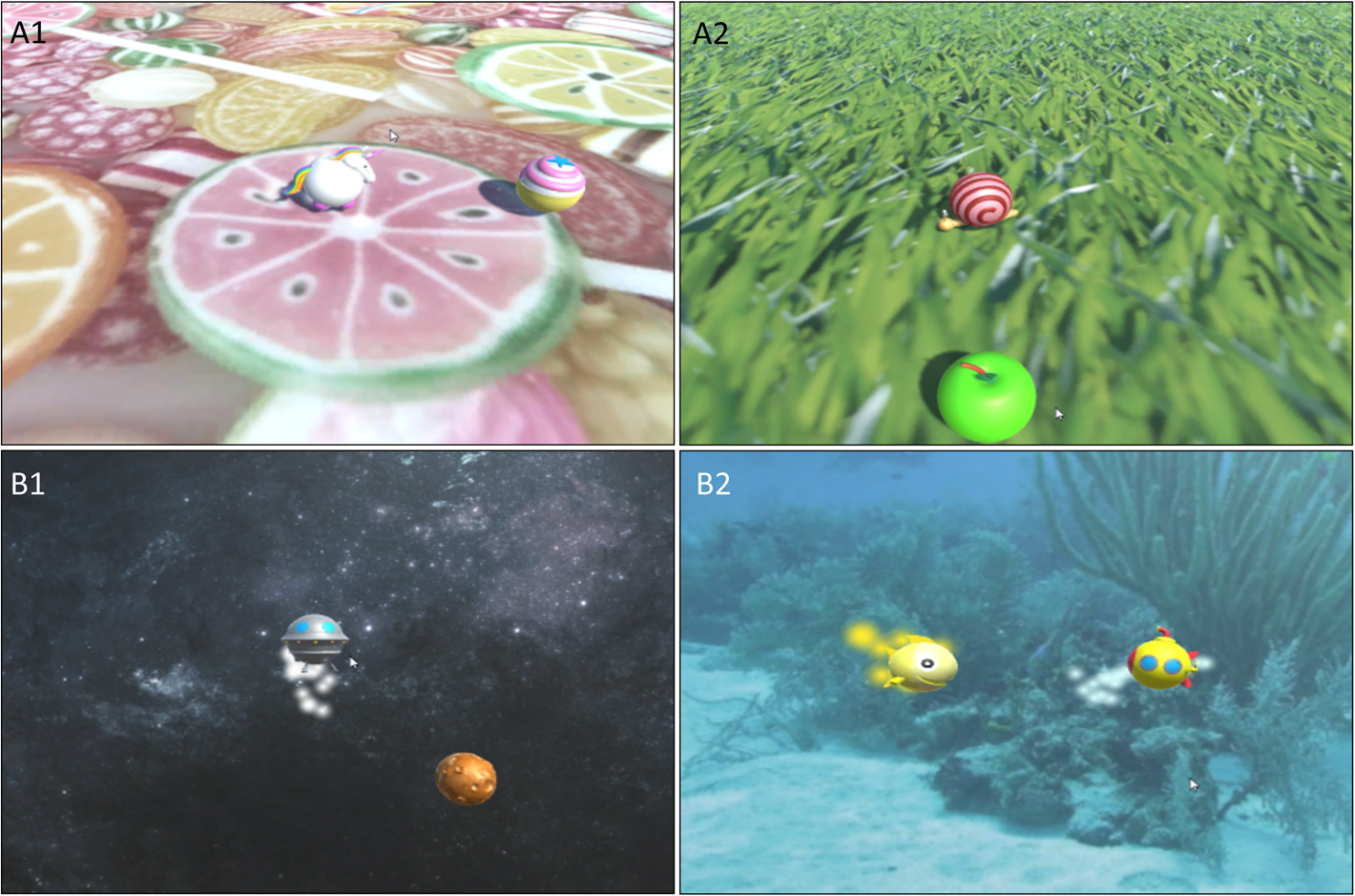
Exergame scenarios displayed on the computer screen. Each exergame version provided two different scenarios. A1 exergame version transversal plane, scenario ‘Unicorn’; A2 exergame version transversal plane, scenario ‘Snail’; B1 exergame version frontal plane, scenario ‘UFO’; B2 exergame version frontal plane, scenario ‘Submarine’

In the exergame, participants had to control an avatar on the screen by changing the position of the hand while being attached to the exoskeleton. Eight different targets, which appear in random order radially around a centre position, had to be reached. The children were instructed to reach the targets ‘using the most direct path and as fast as possible’. The exergame was played in the frontal plane (30 cm or 35 cm in front of the shoulder joint with the small or the large distal module, respectively) and in the transversal plane (10 cm below the shoulder joint with both distal modules) (Fig. 1). Participants performed the reaching movements within and restricted to these planes. Each exergame version provided two scenarios (transversal plane: ‘Unicorn’, ‘Snail’; frontal plane: ‘UFO’, ‘Submarine’) which were shown on the screen (Fig. 2).

### Interventions

Participants of the blocked order group performed all the trials of one exergame version (e.g. in the frontal plane) before switching to the second exergame version (e.g. transversal plane). The participants in the random order group performed the trials of both exergame versions in a pseudo-random order, as we restricted the number of consecutive repetitions of one exergame version to two to keep the contrast between the interventions large. The participants were free to choose between the scenarios for each trial.

The participants trained with the more affected upper limb. If both arms were affected equally, the arm subjectively used as the dominant arm in daily life was chosen for practice.

### Study procedure

**Fig. 3:**
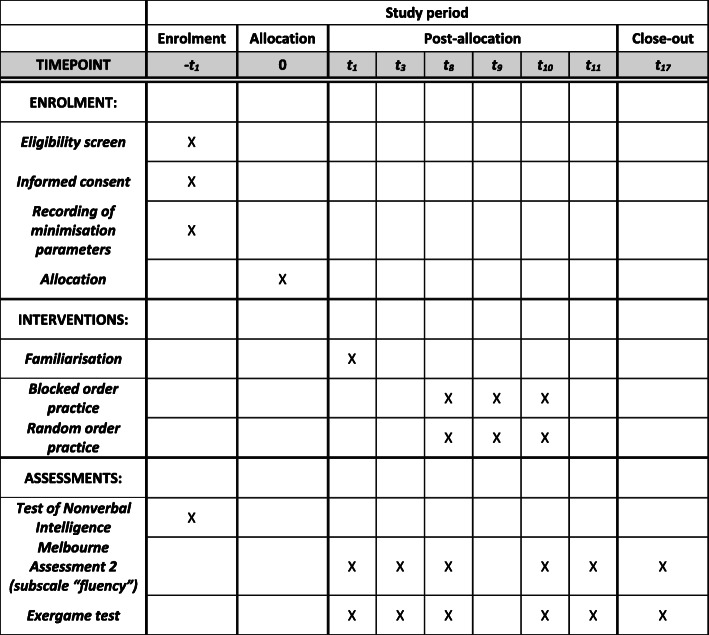
The Standard Protocol Items for Clinical Trials (SPIRIT)-figure: schedule of enrolment, interventions, and assessments. Abbreviations: t = time point

Each participant followed a 3-week schedule. Week 1 was designed as a control week to evaluate potential changes occurring without the study-specific training and compare them with the changes occurring during the practice week. Week 1 contained two sessions of assessments (exergame test and the Melbourne Assessment 2, subscale fluency (MA2_fluency_); for descriptions of the assessments, see below), 2 days apart, without actually practising with the robot. During week 1, as soon as all the descriptors were obtained, group allocation by randomisation through minimisation was performed. Week 2 was the practice week where three practice sessions took place on three consecutive days. The participants practised both exergame versions in either blocked or random order, depending on their group allocation. The number of repetitions was set to 30 per exergame version. Before the first and immediately after the last session, an assessment block consisting of the exergame test and the MA2_fluency_ was performed. The same assessment block was repeated 1 day after the last practice session. Week 3 was the follow-up week with an assessment block 1 week after the last practice session. All sessions (practice and assessments) of the same participant were planned for the same half of the day. Figure 3 shows the SPIRIT Figure, which contains all the study appointments chronologically ordered.

### Outcome measures

We differentiated between three phases of learning: transfer, retention, and acquisition. The transfer was measured with the MA2_fluency,_ which assesses the movement quality in children [28] and is valid, reliable, and responsive in children with cerebral palsy [29]. The sum score of the MA2_fluency_ ranges between zero and 21 points as the seven items are rated with zero to three points. Higher numbers indicate better test performance. An experienced physiotherapist or health scientist administered the MA2 according to the test manual. The MA2_fluency_ was videotaped. An experienced occupational therapist rated the videos of each participant after the child had completed the entire study procedure. The therapist was unaware of the time point when the videos were recorded, as the date and time stamps were deleted.

The retention was measured with data obtained during the exergame tests, which consisted of three trials of each version of the exergame repeated in blocked order. The scenario chosen initially for each exergame version was kept the same throughout all the exergame tests. We chose the parameter ‘number of speed peaks normalised to the actual covered distance’ (nP_norm_). The parameter nP_norm_ is calculated as the number of velocity peaks (i.e. when the difference between a local speed minimum and a local speed maximum exceeds a value of 2.5% of the maximal speed) normalised to the covered distance of the movement path. The parameter nP_norm_ was calculated for each movement (i.e. one value from the centre to the target and one for the return to the centre starting point), leading to 16 data points per trial. As nP_norm_ quantifies movement fluency, it is supposed to reflect a similar construct as the MA2_fluency_. Low nP_norm_ values refer to more fluent movement performance, and nP_norm_ shows very high relative and acceptable absolute reliability (unpublished observations). 

The acquisition was assessed as the change over time during all three practice sessions. To quantify the acquisition, we chose the parameter nP_norm_ calculated for each movement over the exergame trials of the three practice sessions.

#### Primary outcome

For the primary outcome of the trial, we chose the immediate transfer of the practised skill with the sum score of the MA2_fluency_ (i.e. the relative change between the time points immediately before and immediately after the practice session).

#### Secondary outcomes

We chose seven secondary motor learning outcomes. The 1-day delayed transfer and the 1-week-delayed transfer were measured with the MA2_fluency_. The immediate retention, 1-day-delayed retention, 1-week-delayed retention, and the acquisition were measured with the nP_norm_.

Initially, we had planned to investigate the contextual interference effect by performing various statistical analyses to determine immediate and delayed transfer, retention, and acquisition (for the detailed study protocol see [22]). However, it is not recommended to focus on a pilot study on statistical analyses if the power is insufficient [19]; therefore, we presented observations of motor learning in an exploratory manner (Additional file 1).

### Feasibility criteria

We formulated ten criteria a priori and selected appropriate methods to verify the criteria to investigate whether the study protocol would be feasible. We determined the criteria based on Thabane et al. [19], who suggested four categories covering various reasons for conducting a pilot study: process, resources, management, and scientific. We chose and adapted the criteria according to our setting. While we ordered them chronologically in the study protocol [22], here we chose to list them according to their importance, starting with the most important.

1. Are the in- and exclusion criteria specific enough to recruit participants, suitable for this study?
  The main investigator noted the number of participants who were eligible and recruited but not able to perform any of the study appointments due to limitations that were not covered by the in- and exclusion criteria.
2. Is the recruitment rate feasible?
  We defined a fixed recruitment period and calculated the ratio between recruited participants and complete datasets that were obtained within this period.
3. Is it feasible to conduct a future trial with respect to the sample size calculated from the data obtained for the primary outcome?
  Data for calculating a sample size for a future randomised controlled trial could not be provided by the literature as the MA2_fluency_ has not been used solely as an outcome measure before in a comparable study population. Therefore, obtaining data to perform a reliable sample size calculation was another important issue we investigated in this pilot study. We calculated the sample size based on the data obtained from the primary outcome.
4. Is the whole procedure feasible for the participants?
  Participants were regularly asked whether they needed a break or whether they were able to go on with the practice sessions. Based on notes from the lab journal, we evaluated the number of aborted sessions and complete terminations due to the overload of the participants.
5. Is the scheduling procedure feasible?
  The investigator noted the number of appointments, which were not plannable or re-plannable after an unexpected change of schedule.
6. Is the randomisation procedure feasible?
  The number of times in which the randomisation process was not finished on time (i.e. before the first practice sessions) was recorded. We also recorded any issues that occurred during the randomisation procedure (e.g. programme error, unclear where to find the according lists, etc.).
7. Is the handling of the robot feasible?
  Based on notes from the lab journal, we evaluated the technical issues that had led to aborted sessions and trials.
8. Is the processing of a large amount of data feasible?
  The occupational therapists evaluating the primary outcome measure (MA2_fluency_) were asked to report the time generally needed to evaluate six MA2_fluency_ videos of one participant. Furthermore, each exergame trial generated one data file which needed to be processed with a MATLAB script. The appropriate parameters (16 per trial) needed to be exported from the processed data and added in a general data table. The time it took for processing one data file until it was placed in the main data table was recorded for one example data and interpolated for the whole amount of data.
9. Are the outcome measures responsive within this setting?
  The data obtained during the MA2_fluency_ and exergame tests at the time points immediately before and after the practice sessions were used to evaluate internal and external responsiveness. Internal responsiveness measures the ability of an assessment to change over a predefined time period while external responsiveness characterises the extent to which a change measured with the assessment of interest corresponds with the change measured by another tool [30]. We chose this approach to have both, a responsiveness outcome that reflects more the specific context (internal responsiveness) and one that has meaning in a broader range of settings (external responsiveness) [30].
10. Do parallel therapies within the rehabilitation setting influence the results? Assuming that during the first week (normal rehabilitation programme, no additional exergame practice), the change measured with the assessments would be lower than during the practice week, we defined the first week as control week. The data obtained during the MA2_fluency_ and exergame tests at the two time points during the first week were used to evaluate the change within the control week in the whole sample. The participants will have similar therapy schedules during weeks 1 and 2; hence, if no change is measured during week 1, a change during week 2 could at least be partly attributed to the robotic training.

### Target sample size

As the recruitment rate was one of the feasibility criteria, and we also aimed to calculate a sample size for the main study with data obtained from this pilot study, we recruited and enrolled eligible children for 1 year. Based on the number of children undergoing rehabilitation in our centre during the past years (approximately 200 per year) and considering the inclusion and exclusion criteria, we estimated to include 20 children (10 per group) in this pilot study.

### Statistical analyses

Statistical analyses were performed with IBM SPSS Statistics 24. We analysed feasibility criteria 3, 9, and 10 by the following procedures:

Calculating the sample size of a future randomised controlled trial (feasibility criterion 3) was based on the primary outcome, namely the immediate transfer quantified with the MA2_fluency_ data. We used the formula: $n=\frac{{\left({Z}_{\alpha }+{Z}_{\beta}\right)}^2\times 2{\sigma}^2}{{\left({\mu}_1-{\mu}_2\right)}^2}$

here *n*=sample size, *Z*_*α*_=standard normal z value for a significance level *α*=0.05, which is 1.96, and *Z*_*β*_=standard normal z value for the power of 80%, which is 0.84 [31]. The pooled standard deviation of both practice groups is named *σ*; *μ*_1_ and *μ*_2_ are the mean pre-post-intervention difference of the intervention group 1 and intervention group 2, respectively.

The internal responsiveness of the outcome measures (feasibility criterion 9) was determined by calculating the standardised response mean (SRM) for MA2_fluency_ and nP_norm_ between the two assessment time points right before the first and immediately after the last practice session [30]. We performed the analysis for the whole sample, and for the blocked and the random order group separately. The SRM was calculated according to the following formula [32]:

$$ SRM=\frac{{\overline{X}}_{change}}{SD_{change}} $$

where ${\overline{X}}_{change}$is the mean change score and *SD*_*change*_ the standard deviation of the change scores [30, 32]. External responsiveness was determined by calculating a Pearson’s correlation coefficient (*r*) between the pre-post changes of the MA2_fluency_ and the ChARMin parameter nP_norm_ [30]. We would expect a strong negative correlation between the changes in the two measures. In case the data were distributed not normally, we calculated the Spearman correlation.

To investigate the influence of other therapies potentially affecting the outcome (feasibility criterion 10), we used data from the whole sample and determined differences in MA2_fluency_ scores between the two assessment blocks performed in week 1. We used a paired T test (or a Wilcoxon signed-rank test in case of not normally distributed data) and calculated effect sizes (ES) according to the following formula (according to [33]) [32]:

$$ ES=\frac{{\overline{X}}_{change}}{SD_{pooled}} $$

where ${\overline{X}}_{change}$is the mean change and *SD*_*pooled*_ the pooled standard deviation. This ES has been recommended for the use of comparisons with the dependent groups of the same sample.

We interpreted the ES and the SRM according to Cohen’s benchmarks: < 0.2: trivial effect, 0.2–0.5: small effect, 0.5–0.8: moderate effect, > 0.8: large effect [33]. These thresholds have been described to overestimate the SRM, yet as there is no other suitable index for SRM, and since ES and SRM obtained from the chosen formula are comparable [32], we used these thresholds also for SRM.

## Results

**Fig. 4:**
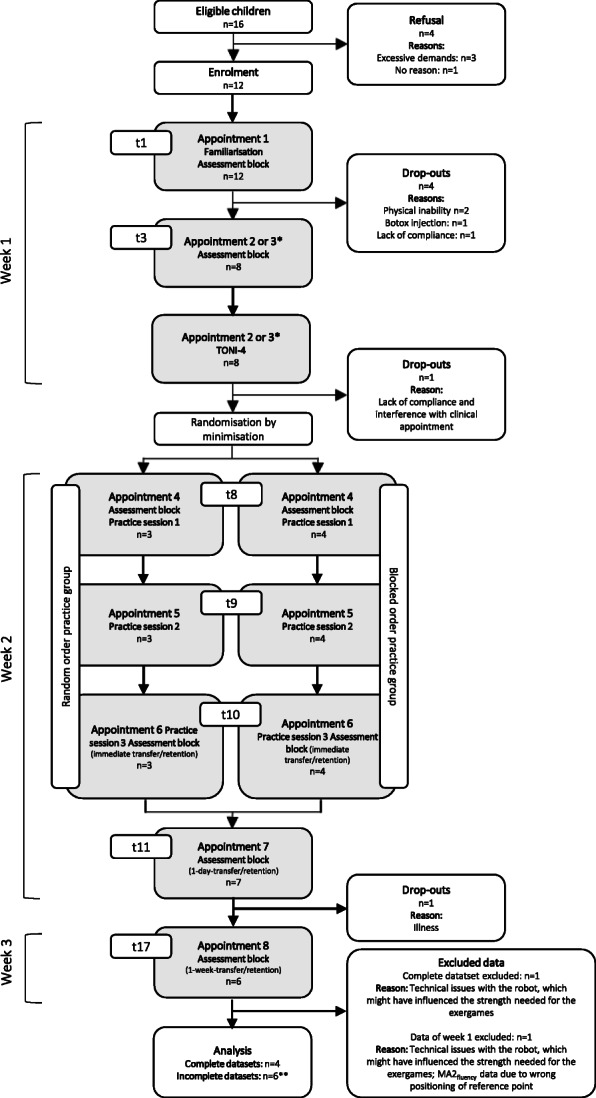
Adherence to the study protocol. *The Test of Nonverbal Intelligence – Fourth edition (TONI-4) was performed at any day between familiarisation (appointment 1) and randomisation. Hence, the TONI-4 appointment was either the second or the third appointment, and the same applies to the second assessment block on t3. **Participant who dropped out after appointment 7 due to illness was included in the analysis with incomplete dataset. Abbreviations: t = time point

**Fig. 5:**
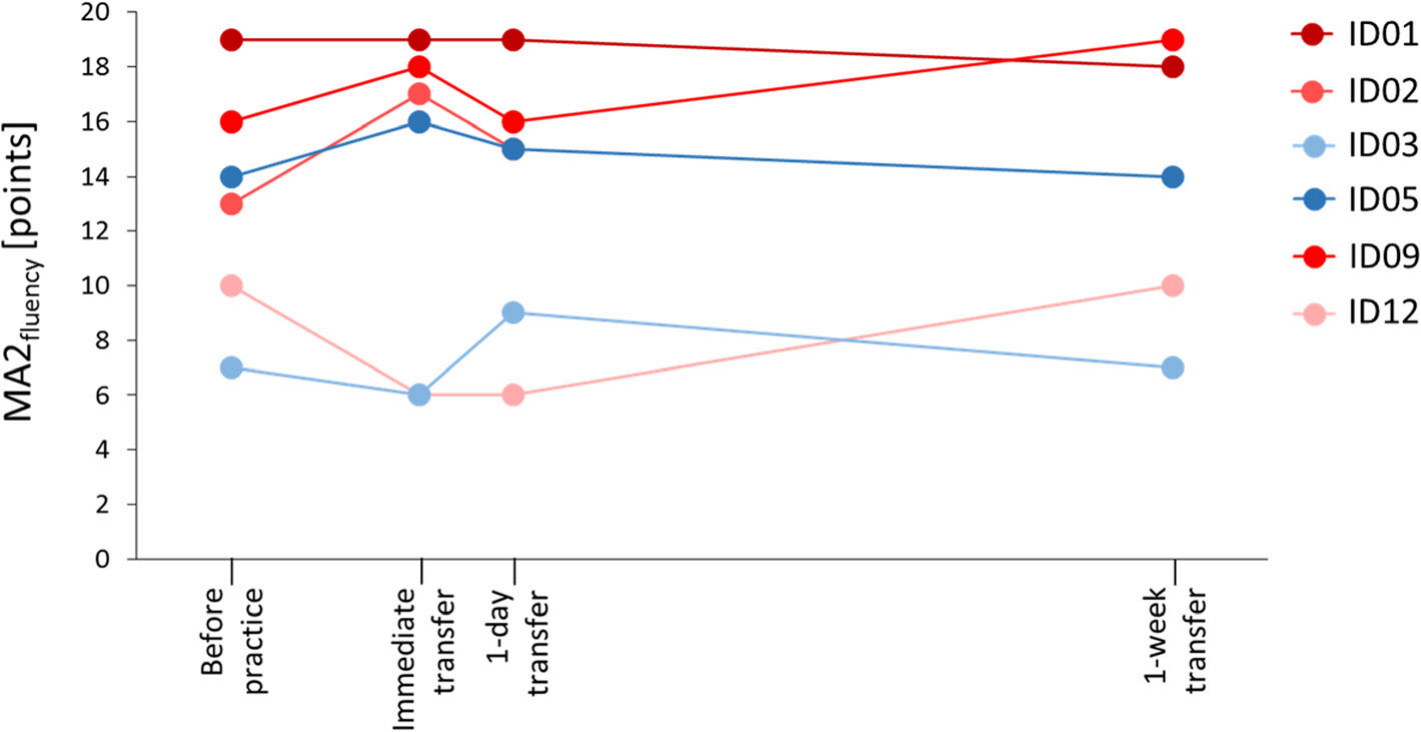
Immediate, 1-day, and 1-week transfer. Displayed are the sum scores of the Melbourne Assessment 2, subscale ‘fluency’ (MA2_fluency_) for each participant at the time points before, immediately after, 1 day and 1 week after practice. Participants are represented with different shades of colours: reddish colours represent blocked practice order, and blueish colours represent random practice order

**Fig 6.**
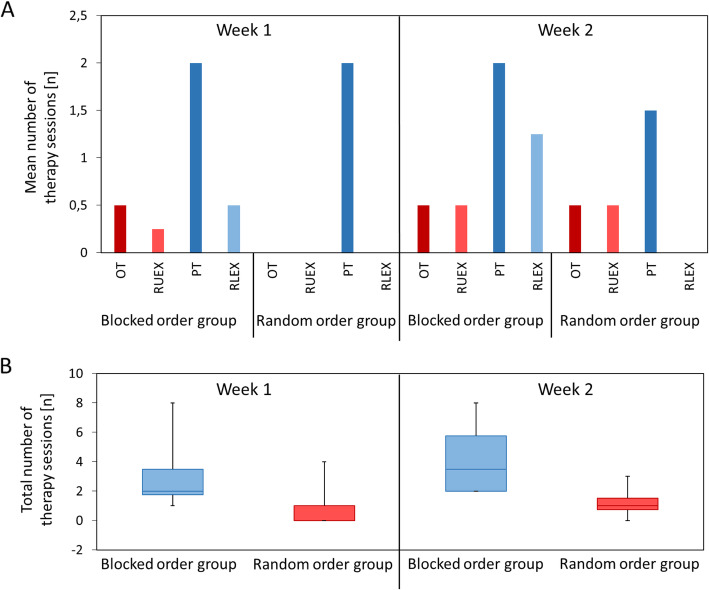
Additional therapies during week 1 and week 2. **A** The mean numbers of the different therapies are displayed for both practice groups and both weeks. **B** Boxplots of the total number of therapies that have taken place during week 1 and week 2 for both practice groups. Abbreviations: OT occupational therapy, RUEX Robotics Upper Extremities, PT Physiotherapy, and RLEX Robotics Lower Extremities

1. Feasibility of inclusion criteria: During familiarisation, two participants were not able to move their more affected arm in the ChARMin robot sufficiently well to play the exergames. One of them had problems moving the arm in the horizontal plane (elbow extension), the other one in both planes.
2. Recruitment rate: Figure 4 shows a flowchart displaying the participants’ adherence to each appointment with reasons for withdrawal or exclusion. Within 1 year, 194 children were admitted to our rehabilitation centre (six of them attended two rehab stays). Only 16 children (8.2%) fulfilled the inclusion criteria and could be informed about the study. Three participants refused to take part due to the demands of the study; one person provided no reason. Twelve participants (75% of the eligible children) were recruited. At familiarisation (first appointment), two participants were not able to perform the exergames due to limited upper limb function, one participant was not compliant to attend the following appointments, and one received upper limb Botox injections after the first appointment, which were unexpectedly planned at short notice. Eight participants (66.7% of recruited participants) attended the second appointment. Here, one participant decided to discontinue the study because of the videotaping. As one participant did not attend the last appointment due to illness, six children (50% of recruited participants) completed the whole procedure. Finally, one dataset had to be excluded due to technical issues with the robot that led to unreliable data. Therefore, we could obtain complete datasets from 41.7% of the recruited participants and one additional dataset with missing data of the final appointment. Table 1 shows the characteristics of the eleven of twelve participants who were enrolled in the study, one participant withdrew from the study during week 1, and the corresponding data were not published.
3. Sample size calculation for a future randomised controlled trial: Based on the results of the primary outcome (MA2_fluency_ mean difference ± standard deviation: blocked order group 0.01 ± 0.30 points, random order group 0.00 ± 0.20 points), we estimated that 15,355 participants should be included when the MA2_fluency_ would be the primary outcome measure. If we would have selected nP_norm_ as the primary outcome measure (nP_norm_ mean difference ± standard deviation: blocked order group −0.09 ± 0.09 normalised speed peaks, random order group −0.14 ± 0.22 normalised speed peaks), 203 participants would be required.
4. Procedure feasible for participants: For 50% of the included participants, the procedure was not feasible. Three participants were not able to complete 30 trials during the three practice sessions (instead of 30/30/30, the number of trials were 20/20/14, 24/24/24, and 28/23/20). Their practice sessions had to be shortened. We paid attention that the sessions did not take longer than 2 h because the burden for the participants would have been too high. That was one reason for aborting the sessions. With time advancing in the practice sessions, we generally noticed a decrease of motivation and concentration and self-reported fatigue, which were also reasons for abortion. One participant prematurely stopped the session due to pain in the shoulder joint.
5. The scheduling procedure proved difficult, as the appointments needed to be planned during the regular inpatient rehabilitation stay. As other therapies had to be considered, it was difficult and time-consuming to plan all the appointments on the same half-day. One participant’s schedule had to be postponed 1 week due to the illness of the participant during the first measurement week. As it was not possible to schedule the follow-up appointment due to the participant’s discharge, the last assessment session was not performed. Furthermore, blinding of the assessors was difficult, because some assessors who assessed the primary outcome measure worked as therapists in the same room and might have noticed how children were practising.
6. The randomisation procedure worked well. Despite the occasional absence of the person primarily in charge of the randomisation by minimisation process, the procedures were always completed in time. In one case, a third person (also not involved in the study) had to operate the randomisation by minimisation programme via telephone instruction by the study nurse, as both the study nurse and her deputy were unavailable.
7. Handling of the robot: One complete dataset and the data of the first week’s appointment of one participant had to be excluded due to a technical issue (weight-support rope was not properly running over a deflection pulley). While this was not a safety issue, it might have influenced the performance during the exergames as there was an increased friction in the movement directions of shoulder flexion and extension. A total of nine single trials had to be repeated due to errors (e.g. when the participant moved too fast, the robot stopped due to safety reasons). Finally, in a few specific configurations, the robot started oscillating, thus reducing the accuracy of the recorded data. The oscillations occur as an interaction between the patient and the robot, i.e. the movement or oscillation of the patient’s arm are amplified by the active joint friction compensation of the robot, leading to an overshoot of the movement. While this occurred in some of the sessions of single patients, the reliable determination of the number of these oscillations was not possible.
8. Amount of data: The therapist assessing the MA2_fluency_ videos had to evaluate a total of 35 videos within one year. Analysing all six videos of one participant cost approximately 35 min. In addition, calculating the parameters of the ChARMin data in Matlab from one practice session (i.e. 30 log files) took approximately 43 min. For three practice sessions, this amounted to more than 2 h. To analyse the exergame test data (6 x 6 log files), approximately 50 min were needed. In case of well-functioning software without any programme errors, approximately 3 h of data processing was needed per participant. Additional time was required occasionally when programme errors occurred (IDs 01, 02, and 03 without errors; ID 04 two errors; IDs 05 and 06 could initially not be processed at all and two software updates were needed to fix the bugs). Overall, approximately 6.5 h per patient were required to analyse the data.
9. Responsiveness of outcome measures: MA2_fluency_ sum-scores per participant are shown before, immediately after, 1 day, and 1 week after the last practice session in Fig. 5. At the immediate transfer, which was our primary outcome, three participants improved (by four, two, and two points), one remained stable, and two participants deteriorated (by minus one and minus four points). Internal responsiveness of the MA2_fluency_ showed a trivial effect for the whole group and the blocked order group (SRM = 0.18 and 0.15, respectively) and a small effect for the random order group (SRM = 0.24). The internal responsiveness analyses of the nP_norm_ data showed that these were moderate to large effects (SRM = −0.84, −0.95, and −0.62 for the whole group, the blocked order group, and the random order group, respectively). External responsiveness: the change in MA2_fluency_ correlated weakly and non-significantly with the change in nP_norm_ for the whole group (*r* = −0.25, *p* = 0.63) and very weak for the blocked group (*r* = 0.03, *p* = 0.97). As in the random group, only two datasets were available, and a correlation could not be calculated.
10. Parallel therapies potentially affecting the outcome: Figure 6 shows the distribution of the different therapies during week 1 (between the two assessment blocks) and during week 2 (between the assessment block before and the one immediately after the practice phase). For one patient, the exergame test as well as the MA2_fluency_ data was not included because of the previously mentioned hardware issue and a mistake in the measurement taken during the MA2 _fluency_ (the reference point where the objects are placed during the test were not equally positioned at both time points). The paired-samples T test between the two assessment time points showed trivial ES and no significant changes neither of the MA2_fluency_ (ES = −0.10, t = −0.74, *p* = 0.50) nor the nP_norm_ data (ES −0.08, t = −0.49, *p* = 0.65).

**Table 1:** Participant characteristics

Participant number	Age [yrs.]	Sex	Weight [kg]	Height [cm]	Diagnose	MACS level	More affected side	Trained side	Practice group
01	11.7	Male	42.0	146.5	Bilateral spastic cerebral palsy	I	Left	Left	Blocked
02	16.2	Female	86.7	161.5	Unilateral spastic cerebral palsy	I	Left	Left	Blocked
03	10.6	Female	43.0	135.0	Bilateral spastic cerebral palsy	III	Right	Right	Random
04	17.1	Male	60.4	188.5	Myelination disorder, ataxic movement disorders	II	Both similar	Left	Random^a^
05	16.8	Female	59.2	167.0	Traumatic brain injury: bilateral, cerebral movement disorder, arms more accentuated (reduced strength and coordination right upper limb)	I	Right	Right	Random
06	16.9	Female	67.1	170.5	Functional hemispherectomy (Rasmussen encephalitis): hemiparesis, arm more accentuated	III	Left	Left	Not allocated^b^
07	14.6	Male	31.9	140.5	Spastic cerebral palsy, legs and right side more accentuated	NA	Right		Not allocated^c^
08	16.8	Female	16.8	57.8	Unilateral spastic cerebral palsy	II	Left	Left	Not allocated^d^
09	6.7	Female	17.2	113.3	Bilateral spastic cerebral palsy	I	Right	Right	Blocked
11	16.2	Male	60.6	164.0	Bilateral spastic cerebral palsy	III	Left	Left	Not allocated ^b^
12	15.1	Male	54.1	168.1	Unilateral spastic cerebral palsy	III	Left	Left	Blocked

One participant (number 10) was not included in the table due to withdrawal of the study including deletion of data

*Abbreviations*: *yrs* years, *kg* kilogramme, *cm* centimetre, *MACS* Manual Ability Classification System, *SD* standard deviation, *NA* not applicable

^a^Excluded after the whole study procedure as data might have been distorted due to technical error

^b^Excluded after familiarisation: physically not able to play the exergame

^c^Excluded after first assessment block (week 1) self-reported compliance not given

^d^Excluded after first assessment block: Botox injection after the first appointment

## Discussion

With this pilot study, we investigated the feasibility of an RCT assessing blocked and random practice order of two versions of an exergame with the ChARMin exoskeleton to improve upper limb functions in children with neuromotor impairments. Of the ten predefined feasibility criteria, we considered six criteria to be partially feasible: in-/exclusion criteria, scheduling, randomisation procedure, handling of the robot, amount of data, influence due to parallel therapies, and four criteria to be not feasible: recruitment rate, participants’ procedure, responsiveness of primary outcome measure, and sample size.

Concerning the feasibility of inclusion criteria, we had selected the MACS level as indicator for the ability to move the upper extremity. For the current study, the MACS proved not suitable as an inclusion criterion, as two participants were not able to perform the movements required for practising the exergames with ChARMin. It follows that the inclusion criteria were not sensitive enough, and therefore, this criterion is only partially feasible. For a future trial, we would recommend to include strength tests. Especially, the shoulder abductors and elbow extensors should have at least a manual muscle score of 3 (movement against gravity over the whole range of motion) [34]. Two participants would have needed the support of ChARMin to perform the exergames in the frontal plane against gravity. Yet, we had decided a priori to refrain from including support by the robot due to standardisation of the interventions. However, ChARMin was designed to train and evaluate also more severely affected patients and the level of weight- and movement support can be adjusted, also for children with more severely affected upper limb abilities [27]. So, another option would be to develop and include a standardised procedure for defining the level of weight support and movement support for patients with different upper limb ability levels and to adapt the support by the robot accordingly to each individual patient.

An eligibility-recruitment rate of 75% and a recruitment and analysable dataset-proportion of 50% can be considered low. With these rates, recruiting would take approximately 1280 years to achieve 15,355 participants (primary outcome measure: MA2_fluency_) or 17 years (nP_norm_ as primary outcome). As a comparison, in a study protocol evaluating the dosage and type of constraint-induced movement therapy in children with cerebral palsy, an eligibility-recruitment rate of 90% is estimated [35]. Another study assessing the efficacy of modified constraint-induced movement therapy also in children with cerebral palsy, screened 76 potential participants and could include 52 of them [36]. Fifty of them attended all the measurements and, accordingly, the recruitment and analysable-dataset proportion was 96% [36]. Different recruitment strategies have been evaluated, yet it has been stated that there is a lack of knowledge about effective recruitment strategies [37]. In our study, any other strategy would have led to the same outcome as we screened and recruited all eligible participants attending our rehabilitation centre. Unfortunately, since the ChARMin robot is not a commercially available device, and a multicentre trial to increase the number of eligible participants would not be possible. A randomised controlled trial requiring these sample sizes is practically impossible within our setting.

The sample size calculation based on the MA2_fluency_ is not feasible to achieve. Calculations based on the exergame test data were lower, yet also not feasible to reach with one device in one centre. It has been recommended to interpret sample size calculations based on pilot studies with caution as estimated treatment effects might be biased due to the low sample size in the pilot study [19]. In fact, estimates of ES based on data obtained from pilot studies have been shown to be insufficiently accurate to be used for the decision-making about whether or not a major trial should be funded [38]. They should, therefore, never be the only criterion to decide about the funding of a trial.

Concerning the feasibility of the study procedure from a participants’ point of view, we had to adjust the procedure several times. The schedule had to be shortened for three participants due to fatigue, low motivation, and in one case due to shoulder pain. Especially in such a heterogeneous study population, it is a challenge to find the number of practice trials sufficient to induce motor learning while being feasible for all participants at the same time. The length and amount of trials of the practice sessions should be individualised according to the participants’ abilities. Yet, in a randomised controlled trial, individually tailoring the interventions could cause an imbalance in terms of the numbers of repetitions. One solution for a future trial would be to increase the number of sessions while simultaneously reducing the number of trials per session. Another approach would be to perform multiple N-of-1 trials, which is indicated when there is substantial uncertainty about the effectiveness of comparable interventions and allows for more individual interventions [39]. Since every participant would serve as their own control, they would practice under both practice orders in a randomised order. Yet, as a motor learning effect would probably be counted as a lasting carry-over effect, a washout period of sufficient length between the two practice periods needs to be considered [39]. This would make it difficult to implement such a design in our setting, where the patients’ length of stay is limited. Hence, practice sessions at home with mobile devices should be discussed for future research.

Scheduling was difficult mainly due to the appointments of the regular rehabilitation schedule, which were treated with higher priority. The rigidity of the study procedure (practice sessions on three consecutive days, follow-up appointments exactly 1 day and 1 week after the last practice session, etc.) negatively affect the possibilities of rescheduling if a participant had missed an appointment. A simpler, more flexible study design would make the (re-)scheduling easier but might affect the learning process in a negative way if conditions would change continuously.

The randomisation procedure worked well, especially since several people were involved in the procedure, absences could be bridged with assigned deputies. Yet, it is recommended to advise a third person a priori to substitute for the deputy in case of his or her absence. A three-person randomisation team would be feasible in our setting.

Technical issues of the current version of ChARMin make the handling of the robot only partially feasible. As the technology is relatively novel, ChARMin may need to undergo some further developments (e.g. preventing the occurrence of movement oscillations or ensuring that the weight-support-rope is running properly) before implementing it in a future trial.

Concerning the evaluation of the outcome measures, evaluating the MA2_fluency_ videos was feasible. Yet processing the ChARMin data (exergame tests and practice) was time consuming. A more automated programme would be needed to handle larger amounts of data.

The MA2_fluency_ was none to moderately responsive when applied as the primary outcome in our study. When looking at the performance of the single participants, after the last practice session, three of them had improved beyond the minimal detectable change (MDC) of 1.84 points for the MA2_fluency_ [29] compared to the onset of practice. One participant did not show a change and two participants deteriorated, one of them beyond the MDC. In comparison, children with cerebral palsy showed a mean improvement of 0.97 points in the MA2_fluency_ after an extensive 8-week upper limb training [29]. That same study obtained an SRM of 1.84 (highly responsive) for the MA2_fluency_ [29]. Yet the intervention was 4.5h per week of uni- or bilateral upper limb training for 8 weeks [29]. Such an intensive intervention is more likely to yield a pre-post-intervention effect compared to our protocol. Indeed, the responsiveness of an outcome measure is determined in a group of patients who have made a true change [40]. As we could not analyse changes due to the intervention in our study, this cannot be verified. The external responsiveness analysis showed a weak correlation between the change measured with MA2_fluency_ and the change measured with nP_norm_ for the whole group. This means that the two measures did not reflect the same construct of measuring change. The internal responsiveness analysis of the nP_norm_ data (all participants included) showed moderate to large SRMs, while the MA2_fluency_ data provided trivial SRMs. The internal responsiveness nicely shows why the correlation of the changes measured with the two measures was weak.

Using a robotic device enables accurate, objective, and sensitive assessment of body functions of children [41]. From this point of view, choosing a robotic device to measure the primary outcome might be recommended. Nevertheless, as we were interested in the transfer and we wanted to measure fluency, we measured fluency outside the robotic device. Therefore, we chose MA2_fluency_ as the primary outcome measure.

Concerning the influence of the parallel therapies, we did not find a change in the MA2_fluency_ between the two time points at week 1. As even the nP_norm_ ES was trivial, in contrast to the large SRMs found during the practice week, it is likely that parallel therapies indeed did not change movement fluency.

### Limitations

There were some limitations in this study. We performed a pilot study in our rehabilitation centre using this specific robotic device. While several feasibility issues can be generalised to other centres, for example, concerning patient recruitment or planning of inpatient appointments, other issues might be specific for our setting, for example, the specific robotic device and its output, restricting generalisability.

We chose to calculate the sample size of a future trial with the primary outcome data. However, determining the sample size based on a meaningful effect such as quantified by the minimal clinically important difference would result in a more appropriate power calculation, including the magnitude of improvement and how patients value the change.

Applying a standardised questionnaire to record how the participants felt (motivation, fatigue, and enjoyment) would have increased data quality and revealed specific descriptors how the participants experienced the study procedure. The Physical Activity Enjoyment Scale Questionnaire would be one example of such a questionnaire [42, 43]. Yet applying an additional outcome measure would have prolonged the assessment protocol even more.

There were also limitations in the study design concerning motor learning aspects. The primary outcome assessor was sometimes present during study appointments. This was unavoidable, as he also worked as a therapist in the same room with other patients. Yet we tried to keep the ratings blinded by making the period between the appointments and the video analysis several weeks long and censoring the videos for the assessment time point. Therefore, we consider this issue as marginal.

Another motor learning issue might have been an insufficient contrast between the interventions (blocked versus random order). The rationale behind the choice of these two interventions is based on the hypothesis that variations of tasks based on different motor programmes result in a contextual interference effect [5]. Different spatial configurations (as it is the case in the current pilot study by moving in different planes) have been suggested to require different motor programmes [4]. This feature has also been chosen in a study evaluating throwing tasks on a horizontal and a vertical target in 6-year-old children [44]. Results showed a superiority of random over blocked practice when comparing the total score during acquisition, at retention, and transfer [44]. However, in addition to the target positions varying in space, the projectiles (four different kinds of balls) were also varied [44]. In our study, the spatial characteristics of the exergames were the only varying factor determining contextual interference. As variations coming from more than one parameter could increase the contextual interference effect [5], we would suggest to increase the contrast between the two study arms for a future trial.

Finally, we could only include six participants in our study, which limits us in making generalisations concerning the motor learning outcomes.

## Conclusion

We could show that it is not feasible to perform a large RCT when using the design as evaluated in this pilot study. The main reasons are the low recruitment rate, the demanding study procedures, and the large sample size that would be needed for the main trial. We made several suggestions on how to improve the study design and discussed alternatives such as n-of-1 trials.

Studies on whether the contextual interference effect in children with neuromotor disorders exists are still needed, as this could guide future research and clinical treatment of this vulnerable patient group.

## Supplementary Information

**Additional file 1.** Portable document format (.pdf). Title: Observations of motor learning. This document contains a summary of observations (including graphs) related to motor learning, resulting from the data obtained during this pilot study.

## Data Availability

The datasets used and/or analysed during the current study are available from the corresponding author on reasonable request.

## Abbreviations

CONSORT: Consolidated Standards of Reporting Trials
ES: Effect size
MACS: Manual Ability Classification System
MDC: Minimal detectable change
MA2_fluency_: Melbourne Assessment 2, subscale fluency
NA: Not applicable
nP_norm_: Number of speed peaks normalised to the actually covered distance
OT: Occupational therapy
PT: Physiotherapy
r: Pearson’s correlation coefficient
RCT: Randomised controlled trial
RLEX: Robotics lower extremities
RUEX: Robotics upper extremities
SD: Standard deviation
SPIRIT: Standard Protocol Items: Recommendations for Interventional Trials
SRM: Standardised response mean
TONI-4: Test of Nonverbal Intelligence – Fourth edition

## References

[1] Krakauer JW (2006). Motor learning: its relevance to stroke recovery and neurorehabilitation. Curr Opin Neurol.

[2] Muratori LM, Lamberg EM, Quinn L, Duff SV (2013). Applying principles of motor learning and control to upper extremity rehabilitation. J Hand Ther.

[3] Schmidt RA, Lee TD. Motor learning and performance. From principles to application. 5th ed. Champaign: Human Kinetics Publishers; 2014.

[4] Bernstein NA (1967). The co-ordination and regulation of movements.

[5] Magill RA, Hall KG (1990). A review of the contextual interference effect in motor skill acquisition. Hum Mov Sci.

[6] Shea JB, Morgan RL (1979). Contextual interference effects on the acquisition, retention, and transfer of a motor skill. J Exp Psychol Hum Learn Mem.

[7] Shea JB, Zimny S, Magill RA (1983). Context effects in memory and learning movement information. Memory and control of action.

[8] Lee TD, Magill RA, Goodman D, Wilberg R, Franks I (1985). Can forgetting facilitate skill acquisition?. Differing perspectives in motor learning, memory, and control.

[9] Poto CC (1988). How forgetting facilitates remembering: an analysis of the contextual interference effect in motor learning.

[10] Thürer B, Gedemer S, Focke A, Stein T. Contextual interference effect is independent of retroactive inhibition but variable practice is not always beneficial. Front Hum Neurosci. 2019;13. 10.3389/fnhum.2019.00165. 10.3389/fnhum.2019.00165 PMC6557302 31213998

[11] Shewokis PA, Del Rey P, Simpson KJ (1998). A test of retroactive inhibition as an explanation of contextual interference. Res Q Exerc Sport.

[12] Brady F (2004). Contextual interference: a meta-analytic study. Percept Mot Skills.

[13] Graser JV, Bastiaenen C, van Hedel H. The role of the practice order: a systematic review about contextual interference in children. PLoS One. 2019;14(1):e0209979. 10.1371/journal.pone.0209979. 10.1371/journal.pone.0209979 PMC6342307 30668587

[14] Aurich-Schuler T, van Hedel HJA, Labruyère R (2018). Roboterunterstützte Lokomotionstherapie bei Kindern in der Neuroreha. Neuroreha.

[15] van Hedel HJA, Lieber J, Ricklin S, Meyer-Heim A (2017). Die praktische Anwendung von Exergames und virtueller Realität in der pädiatrischen Rehabilitation. Neuroreha.

[16] Gerber CN, Kunz B, van Hedel HJA (2016). Preparing a neuropediatric upper limb exergame rehabilitation system for home-use: a feasibility study. J Neuroeng Rehabil.

[17] Duret C, Grosmaire A-G, Krebs HI (2019). Robot-assisted therapy in upper extremity hemiparesis: overview of an evidence-based approach. Front Neurol.

[18] Prado MTA, Gonçalves Luiz Fernani DC, Dias da Silva T, Smorenburg ARP, de Abreu LC, Bandeira de Mello Monteiro C (2017). Motor learning paradigm and contextual interference in manual computer tasks in indivisuals with cerebral palsy. Res Dev Disabil.

[19] Thabane L, Ma J, Chu R, Cheng J, Ismaila A, Rios LP, Robson R, Thabane M, Giangregorio L, Goldsmith CH. A tutorial on pilot studies: the what, why and how. BMC Med Res Methodol. 2010;10(1). 10.1186/1471-2288-10-1. 10.1186/1471-2288-10-1 PMC2824145 20053272

[20] Eldridge SM, Chan CL, Campbell MJ, Bond CM, Hopewell S, Thabane L, Lancaster GA, PAFS consensus group. CONSORT 2010 statement: extension to randomised pilot and feasibility trials. BMJ (Clinical research ed.). 2016;355(i5239). 10.1136/bmj.i5239. 10.1136/bmj.i5239 PMC5076380 27777223

[21] Chan A-W, Tetzlaff JM, Altman DG (2013). SPIRIT 2013 Statement: defining standard protocol items for clinical trials. Ann Intern Med.

[22] Graser JV, Bastiaenen C, Keller U, van Hedel H. Contextual interference in children with brain lesions: protocol of a pilot study investigating blocked vs. random practice order of an upper limb robotic exergame. Pilot and feasibility studies. 2020;6:156. 10.1186/s40814-020-00694-y. 10.1186/s40814-020-00694-y PMC7560185 33072397

[23] Eliasson A-C, Krumlinde-Sundholm L, Rösblad B, Beckung E, Arner M, Ohrvall A-M (2006). The Manual Ability Classification System (MACS) for children with cerebral palsy: scale development and evidence of validity and reliability. Dev Med Child Neurol.

[24] Bohannon RW, Smith MB (1987). Interrater reliability of a modified Ashworth scale of muscle spasticity. Phys Ther.

[25] Altman DG, Bland JM (2005). Treatment allocation by minimisation. Br Med J.

[26] Ritter N, Kilinc E, Navruz B, Bae Y (2011). Test Review: L. Brown, R. J. Sherbenou, & S. K. Johnsen ‘Test of Nonverbal Intelligence-4’ (Toni-4). Austin, TX--PRO-ED, 2010. J Psychoeduc Assess.

[27] Keller U, Van Hedel HJA, Klamroth-Marganska V, Riener R (2016). ChARMin: the first actuated exoskeleton robot for pediatric arm rehabilitation. IEEE/ASME Trans Mechatronics.

[28] Randall M, Johnson L, Reddihough D. The Melbourne Assessment 2. Available from: https://www.rch.org.au/melbourneassessment/. Cited 2019 Mar 29

[29] Wang T-N, Liang K-J, Liu Y-C, Shieh J-Y, Chen H-L (2017). Psychometric and clinimetric properties of the Melbourne Assessment 2 in children with cerebral palsy. Arch Phys Med Rehabil.

[30] Husted JA, Cook RJ, Farewell VT, Gladman DD (2000). Methods for assessing responsiveness: a critical review and recommendations. J Clin Epidemiol.

[31] Hickey GL, Grant SW, Dunning J, Siepe M (2018). Statistical primer: sample size and power calculations — why, when and how?. Eur J Cardio-Thoracic Surg.

[32] Middel B, van Sonderen E. Statistical significant change versus relevant or important change in (quasi) experimental design: some conceptual and methodological problems in estimating magnitude of intervention-related change in health services research. Int J Integr Care. 2002;2(4). 10.5334/ijic.65. 10.5334/ijic.65 PMC1480399 16896390

[33] Cohen J (1988). Statistical power analysis for the behavioral sciences. 2nd editio.

[34] Kendall McCreary E, Peterson Kendall F, Geise PP (1993). Muscles: testing and function.

[35] Landesman Ramey S, DeLuca S, Stevenson RD, Case-Smith J, Darragh A, Conaway M. Children with Hemiparesis Arm and Movement Project (CHAMP): protocol for a multisite comparative efficacy trial of paediatric constraint-induced movement therapy (CIMT) testing effects of dosage and type of constraint for children with hemiparetic cerebra. BMJ Open. 2019;9(1). 10.1136/bmjopen-2018-023285 PMC6340418 30782701

[36] Ramey SL, DeLuca S, Stevenson RD, Case-Smith J, Darragh A, Conaway M. Children with Hemiparesis Arm and Movement Project (CHAMP): protocol for a multisite comparative efficacy trial of paediatric constraint-induced movement therapy (CIMT) testing effects of dosage and type of constraint for children with hemiparetic cerebral palsy. BMJ open. 2019;9(1):e023285. 10.1136/bmjopen-2018-023285. 10.1136/bmjopen-2018-023285 PMC6340418 30782701

[37] Treweek S, Mitchell E, Pitkethly M, Cook J, Kjeldstrøm M, Johansen M, et al. Strategies to improve recruitment to randomised controlled trials (Review). Cochrane Database Syst Rev. 2010;1(MR000013). 10.1002/14651858.MR000013.pub4 20091668

[38] Treweek S, Pitkethly M, Cook J, Fraser C, Mitchell E, Sullivan F, Gardner H. Strategies to improve recruitment to randomised trials. Cochrane Database of Systematic Reviews. John Wiley and Sons Ltd. 2018. 10.1002/14651858.MR000013.pub6. 10.1002/14651858.MR000013.pub6 PMC7078793 29468635

[39] Kravitz R, Naihua D, Sunita V, Jiang L, RL K, Duan N (2014). The DEcIDE Methods Center N-0f-1 Guidance Panel. Introduction to N-of-1 trials: indications and barriers. Design and implementation of N-of-1 trials: a user’s guide.

[40] Carter R, Lubinsky J, Domholdt E (2005). Rehabilitation research: principles and applications.

[41] Gilliaux M, Lejeune TM, Detrembleur C, Sapin J, Dehez B, Selves C, Stoquart G (2014). Using the robotic device REAplan as a valid, reliable, and sensitive tool to quantify upper limb impairments in stroke patients. J Rehabil Med.

[42] Latorre Román PÁ, García Pinillos F, Navarro Martínez AV, Izquierdo RT (2014). Validity and reliability of Physical Activity Enjoyment Scale questionnaire (PACES) in children with asthma. J Asthma.

[43] Boffoli N, Foley JT, Gasperetti B, Yang SP, Lieberman L (2011). Enjoyment levels of youth with visual impairments playing different exergames. Insight Res Pract Vis Impair Blind.

[44] Granda Vera J, Montilla MM (2003). Practice schedule and acquisition, retention, and transfer of a throwing task in 6-yr.-old children. Percept Mot Skills.

