# Supplementary material for: Contextual interference in children with brain lesions: a pilot study investigating blocked vs. random practice order of an upper limb robotic exergame
Source: Pilot Feasibility Stud. 2021 Jun 25;7:135. doi: 10.1186/s40814-021-00866-4 (PMC8228977; doi:10.1186/s40814-021-00866-4)
Supplement: Supplementary file 1 — Additional file 1. Portable document format (.pdf). Title: Observations of motor learning. This document contains a summary of observations (including graphs) related to motor learning, resulting from the data obtained during this pilot study. [file 40814_2021_866_MOESM1_ESM.pdf]

Contextual interference in children with brain lesions: a pilot study investigating blocked vs. random practice order of an upper limb robotic exergame

Judith V Graser, Caroline HG Bastiaenen, Anja Gut, Urs Keller, Hubertus JA van Hedel

Observations of motor learning

As there were only six datasets available, we present some aspects about motor learning worth discussing in an exploratory manner in this supplementary file.

Transfer

A visualisation of the Melbourne Assessment 2, subscale Fluency ($MA2_{fluency}$) sum-scores per participant at the time points before, immediately after, one day and one week after the last practice session is displayed in Figure A1 (equal to Figure 5 in the main manuscript).

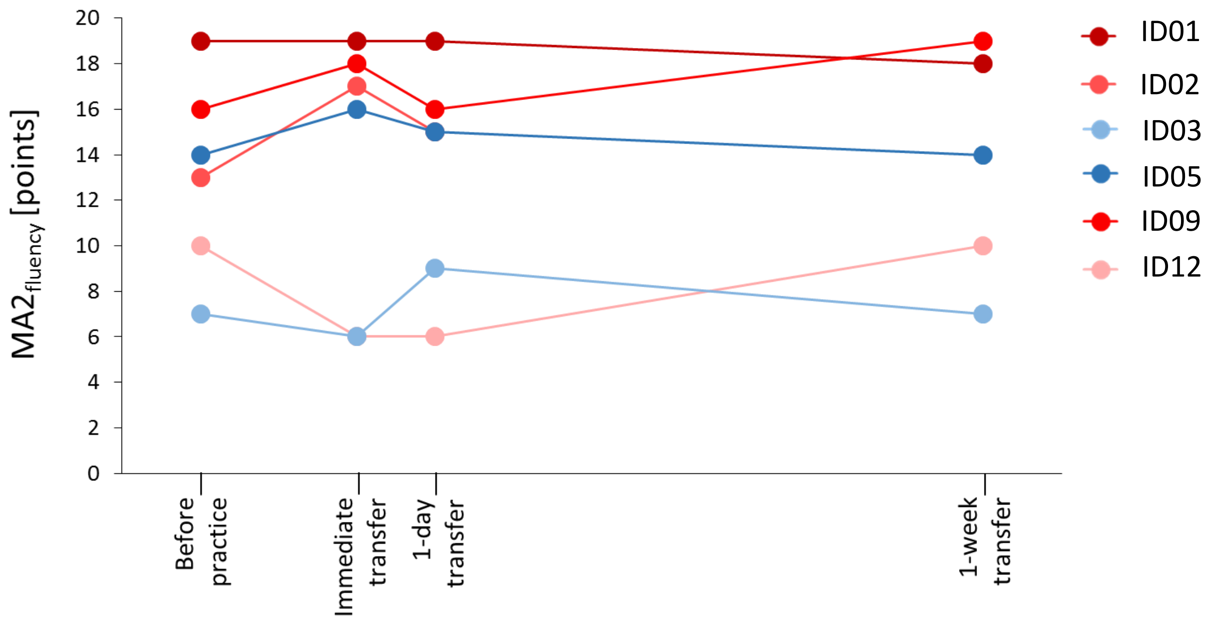

Fig A1. Immediate, 1-day and 1-week transfer. The Melbourne Assessment 2 subscale Fluency sum scores ($MA2_{fluency}$; Y-axis) are plotted for each time point (X-axis) for each participant. Participants are represented with different shades of colours, reddish colours represent blocked practice order, blueish colours represent random practice order. (This Figure is equal to Figure 5 in the main manuscript).

At the immediate transfer, which we initially chose as primary outcome time point, three participants improved beyond the minimal detectable change (MDC) of 1.84 points for $MA2_{fluency}$ (1). However, one participant did not show a change and two participants

deteriorated, one of them beyond the MDC. It is noteworthy, that at least some of the participants showed a substantial change in performance of the $MA2_{fluency}$ within such a short time. In comparison, children with cerebral palsy showed a mean improvement of 0.97 points in the $MA2_{fluency}$ after an extensive eight-weeks upper limb training (1).

Retention

Data obtained during the exergame tests and used for retention are displayed in Figure A2.

At immediate retention, four participants improved (-4.3, -3.3, -7.3 and -2.0 velocity peaks per movement, normalised to the actual distance). The other two showed slight deteriorations (0.5 and 0.9 velocity peaks per movement, normalised to the actual distance). These two participants started with lower performance levels (i.e. higher numbers of peaks) compared to the four participants who improved during practice. Yet, they improved at delayed retention.

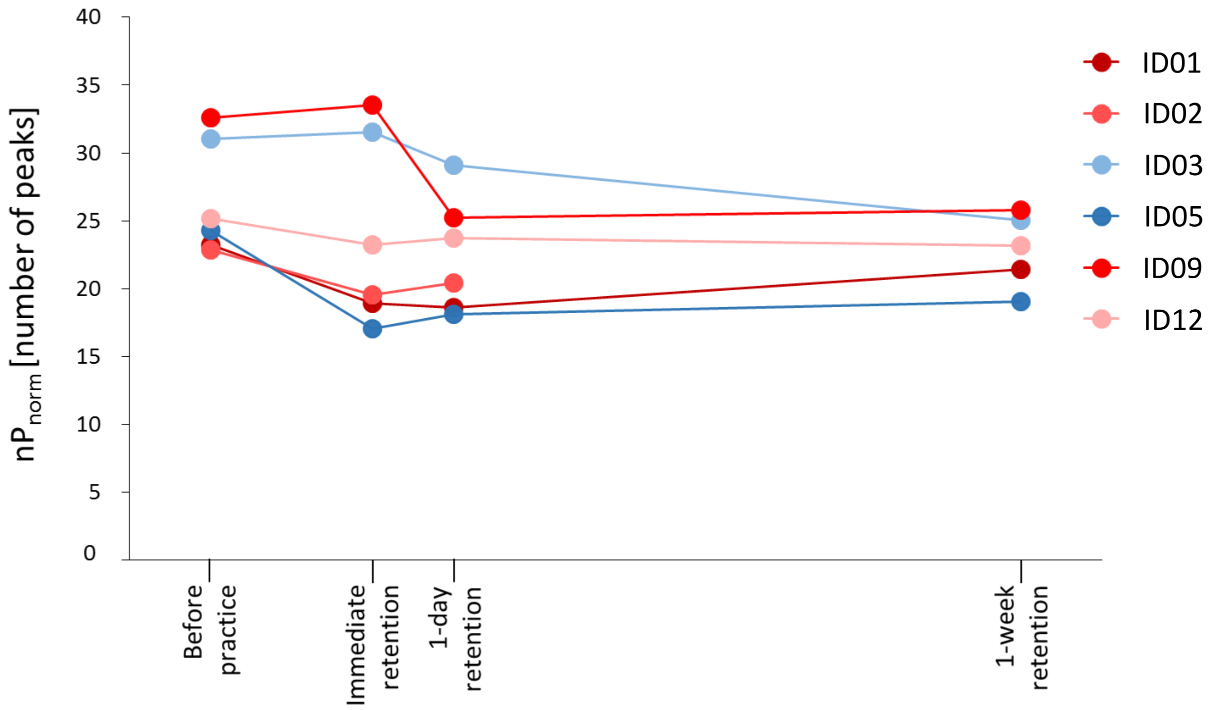

Fig A2. Immediate, 1-day, and 1-week retention. The mean numbers of velocity peaks normalised to the actual distance (nP_{norm}) over one game test, i.e. three trials on the horizontal and three trials on the frontal plane (Y-axis) are plotted for each time point (X-axis) for each participant. Participants are represented with different shades of colours, reddish colours represent blocked practice order, blueish colours represent random practice order.

These 'groups' were not reflecting the practice groups. We assume that the slight deteriorations in the two participants might reflect normal trial-to-trial variability or they might have temporarily decreased their performance at the end of the practice period due to,

for example, fatigue. Indeed, at the one-day retention, their performance improved, which we could explain as a sort of recovery (e.g. from fatigue). This could be an indication that retention test should be conducted after a break to allow participants to recover from the intensive practice.

When comparing the data at one-week retention with the data from before the practice sessions, all participants (with the exception of one participant who did not attend the one-week retention test) improved their performance by -1.8 to -6.8 velocity peaks.

Acquisition

Figure A3 shows the plotted data of all trials and participants with a linear trend-line over the course of the three practice sessions. No general trend can be observed. Some of the participants seem to deteriorate over the three sessions while some seem to improve. Some participants show fluctuations during or between the sessions, while others perform on a relatively steady level.

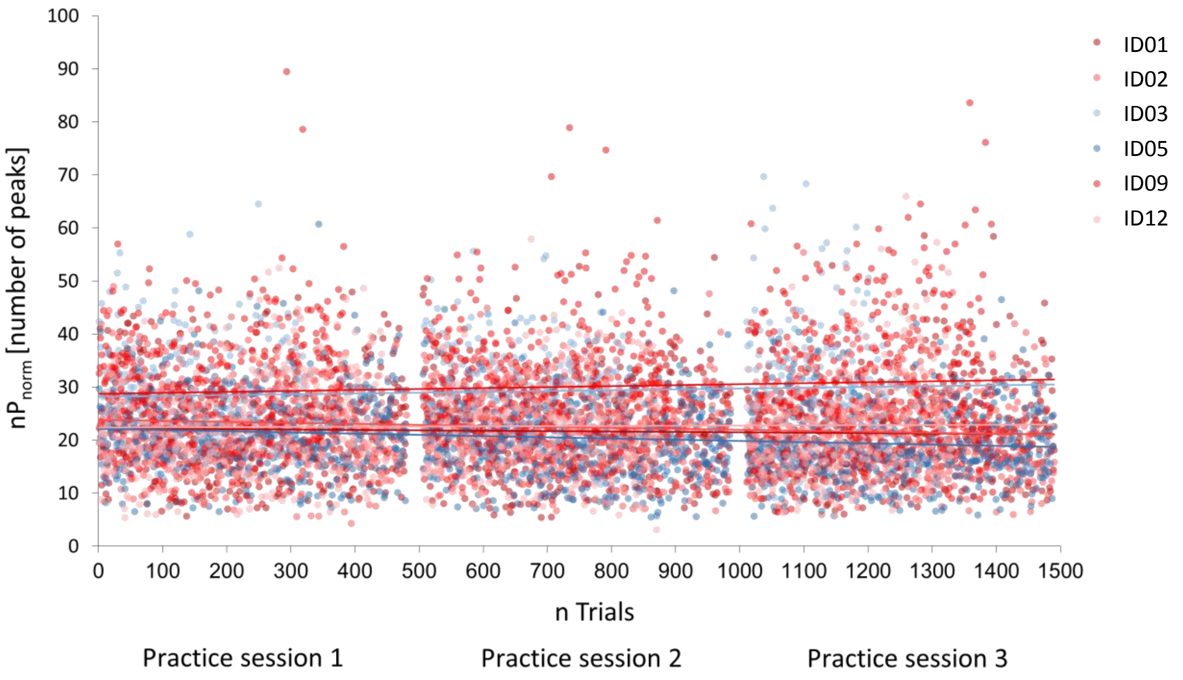

Fig A3. Acquisition: performance during the practice phase. The number of velocity peaks normalised to the actual distance (nP_{norm}; Y-axis) are plotted (participants are represented with different shades of colours, reddish colours represent blocked practice order, blueish colours represent random practice order.) with linear trend lines for each participant (according opaque colours) over all trials of all three practice sessions (X-axis).

This widespread fluctuation in performance during acquisition within each practice session and each individual participant is eye catching. There are some general factors like motivation

(2) or fatigue (3) influencing variability in motor performance and learning. In our study, we identified also some movement-related factors. Firstly, within each trial, participants head for eight targets and move each time back to the centre point meaning that all 16 movements point in another direction. Depending on a child's motor impairments, movement experience, and preferences, one movement direction might be easier to be performed fluently compared to another one. Secondly, the participants might have preferences for moving in the horizontal or frontal plane. Such aspects could be partly responsible for the widespread performance within each participant.

References

1. Wang T-N, Liang K-J, Liu Y-C, Shieh J-Y, Chen H-L. Psychometric and clinimetric properties of the Melbourne Assessment 2 in children with cerebral palsy. Arch Phys Med Rehabil. 2017;98(9):1836–41.
2. Wulf G, Lewthwaite R. Optimizing Performance through Intrinsic Motivation and Attention for Learning: The OPTIMAL theory of motor learning. Psychon Bull Rev. 2016;22(6):1–35.
3. Branscheidt M, Kassavetis P, Anaya M, Rogers D, Huang HD, Lindquist MA, et al. Fatigue induces long-lasting detrimental changes in motor-skill learning. Elife. 2019;8:e40578:1–25.
